# The role of landscape composition and heterogeneity on the taxonomical and functional diversity of Mediterranean plant communities in agricultural landscapes

**DOI:** 10.1371/journal.pone.0238222

**Published:** 2020-09-16

**Authors:** Joana Cursach, Juan Rita, Carmelo Gómez-Martínez, Carles Cardona, Miquel Capó, Amparo Lázaro

**Affiliations:** 1 Research Group on Plant Biology under Mediterranean Conditions, Laboratory of Botany, Department of Biology, University of the Balearic Islands, Palma, Balearic Islands, Spain; 2 Global Change Research Group, Mediterranean Institute for Advanced Studies (UIB-CSIC), Esporles, Balearic Islands, Spain; 3 Interdisciplinary Ecology Group, Laboratory of Botany, Department of Biology, University of the Balearic Islands, Palma, Balearic Islands, Spain; 4 Centre Forestal de les Illes Balears, Institut Balear de la Natura, Conselleria de Medi Ambient i Territori, Palma, Balearic Islands, Spain; Seoul National University, REPUBLIC OF KOREA

## Abstract

The expansion of agriculture is a major driver of biodiversity loss worldwide, through changes generated in the landscape. Despite this, very little is still known about the complex relationships between landscape composition and heterogeneity and plant taxonomical and functional diversity in Mediterranean ecosystems that have been extensively managed during millennia. Although according to the Intermediate Disturbance Hypothesis (IDH) plant richness might peak at intermediate disturbance levels, functional diversity might increase with landscape heterogeneity and decrease with the intensity of disturbance. Here, we evaluated the associations of landscape composition (percentage of crops) and heterogeneity (diversity of land-cover classes) with plant taxonomical diversity (richness, diversity, evenness), local contribution to beta diversity, and functional diversity (functional richness, evenness, divergence and dispersion) in 20 wild *Olea europaea* communities appearing within agricultural landscapes of Mallorca Island (Western Mediterranean Basin). In accordance with the IDH, we found that overall plant richness peaked at intermediate levels of crops in the landscape, whereas plant evenness showed the opposite pattern, because richness peak was mainly related to an increase in scarce ruderal species. Plant communities surrounded by very heterogeneous landscapes were those contributing the most to beta diversity and showing the highest functional richness and evenness, likely because diverse landscapes favour the colonization of new species and traits into the communities. In addition, landscape heterogeneity decreased functional divergence (i.e., increased trait overlap of dominant species) which may enhance community resilience against disturbances through a higher functional redundancy. However, a large extent of agriculture in the landscape might reduce such resilience, as this disturbance acted as an environmental filter that decreased functional dispersion (i.e, remaining species shared similar traits). Overall, our study highlights the importance of considering several indices of taxonomical and functional diversity to deeply understand the complex relationships between ecosystems functions and landscape context.

## Introduction

Maintaining high levels of biodiversity is crucial for the stability of communities against disturbances [1, 2]. However, land-use changes, and especially the expansion and intensification of agriculture, strongly threaten the biodiversity worldwide [3–5]. Insular Mediterranean landscapes have been strongly shaped through millennia by human use, usually resulting in complex mosaics of traditional non-intensive crop cultivation interspersed with wild vegetation [6], and are more susceptible to biodiversity loss than mainland ones [7]. Land-use changes influence landscape composition, i.e. type and extent of habitats contained within the landscape, as well as landscape heterogeneity, i.e. the diversity of habitats in the landscape, which in turn may directly affect both plant taxonomical and functional diversity.

Several studies have shown that changes in landscape composition and, particularly, the loss of natural and semi-natural habitats, have a negative effect on plant taxonomical diversity [8–11]. Nevertheless, the relationships between disturbance and diversity might not be always linear. Indeed, the Intermediate Disturbance Hypothesis (IDH) predicts that species diversity will be highest at intermediate levels of disturbance because there will be a balance between competitive exclusion and the establishment of dominant species [12, 13]. IDH has been assessed for diverse types of disturbances and in different habitats [14–19]. However, the empirical support to IDH is still inconclusive, as a recent review showed that only the 46% of studies (22 out of 48) in terrestrial ecosystems supported it, most of them in upland sites [20]. Moreover, it has been shown that the conformity to IDH may depend on other factors, such as the environmental stress of communities [19].

Land-use changes also affect the heterogeneity of habitats in the landscape, which may drive to cascading effects on plant diversity because species distribution and composition is largely environmental determined [21, 22]. A more heterogeneous landscape implies an increase in habitat diversity and thus, in ecological niches, which in turn might positively influence species richness [23, 24]. Indeed, previous studies have reported that plant richness increases with landscape heterogeneity as measured with different indices [25–27]. Similarly, previous studies have shown that beta diversity of bird communities increases [28] and that pollinator visits to crops stabilize [29] with landscape heterogeneity in Mediterranean agricultural landscapes.

Traditionally, changes in biodiversity have been assessed by species richness and evenness; however, to evaluate how plant communities respond to environmental changes is crucial to take into account the diversity of functional traits they hold [30], that is, the biological attributes (physiological, structural, and behavioural) that influence the performance of organisms [31]. Several indices have been proposed to capture the different aspects of functional diversity (i.e., functional richness, evenness, divergence and dispersion; [32, 33]), which have been shown to respond differently to disturbance gradients. For instance, Malavasi et al. [9] showed that functional diversity (i.e., the amount of species distinctiveness in a community expressed as species richness multiplied by funcional evenness and mean trait dispersion) and evenness (i.e., the homogeneity in the abundance distribution of traits in a community) decreased respectively with the increase in artificial areas and fragmentation in Mediterranean coastal dune ecosystems. Besides, Rochas-Santos et al. [34] reported that functional richness of tree reproductive traits decreased and functional divergence (i.e., clustering in the abundance distribution of traits in a community) increased as the amount of natural habitat (forest cover) decreased in the Atlantic rainforest flora. In general, changes in the landscape that affect taxonomical richness and species composition might influence functional diversity. However, functional indices that take into account the relative abundance of species, such as functional evenness and divergence, might be more sensitive than functional richness to disturbance [33]. On the other hand, functional dispersion (i.e., mean distance in multidimensional trait space of individual species to the centroid of all species) might help to understand changes in functional diversity that are independent of species richness [33].

In this study, we assessed the effects of landscape heterogeneity and composition on both the taxonomical and functional diversity of plant communities in Mallorca (western Mediterranean Basin). For this, we selected 20 shrubland communities dominated by wild *Olea europaea* across agricultural landscapes to evaluate how landscape composition (percentage of crops and natural areas) and landscape heterogeneity (the diversity of land-cover classes) influenced: 1) plant taxonomical diversity (total richness and richness of ruderals vs. non-ruderals, Shannon’s diversity, and evenness); 2) the local contribution to beta diversity of plant communities (how unique the communities were in terms of species); 3) overall plant functional diversity (estimated as functional richness, functional evenness, functional divergence and functional dispersion). Generally, we hypothesized that taxonomical diversity would be highest at intermediate levels of percentage of crops in the landscape, in accordance with the IDH, and that both local contribution to beta diversity and overall functional diversity would increase with increasing landscape heterogeneity, especially when measured with indices that are influenced by species abundance.

## Material and methods

### Study area and sites

We carried out the study across Mallorca Island (39°37’N, 2°59’E), the largest island within the Balearic Islands Archipelago, Spain. From the biogeographical point of view, Mallorca belongs to the Eastern Balearic Islands, which include the Gymnesians and adjacent islets [35], and it is characterized by a significantly diverse flora: 1445 taxa -125 of which are endemics-, and a greater representation of Tyrrhenian species compared to Western Balearic Islands [35]. The Balearics are characterized by a Mediterranean climate, with warm summers (maximum mean monthly records exceeding 30 ºC) and mild winters (minimum mean monthly temperatures above 5 ºC) and average annual accumulated precipitation of 585 mm [36]. The Balearics have mainly a thermo-Mediterranean climate, with meso- and supra-Mediterranean climates in the mountains of Mallorca. Ombroclimates range from humid to semi-arid, although the most common are sub-humid and dry. The predominant forest vegetation consists of the evergreen forests, woodlands and sclerophyllous shrublands dominated by *Quercus ilex*, *Pinus halepensis*, *Olea europaea*, *Pistacia lenticus* and *Juniperus turbinata* [37].

**Fig 1:**
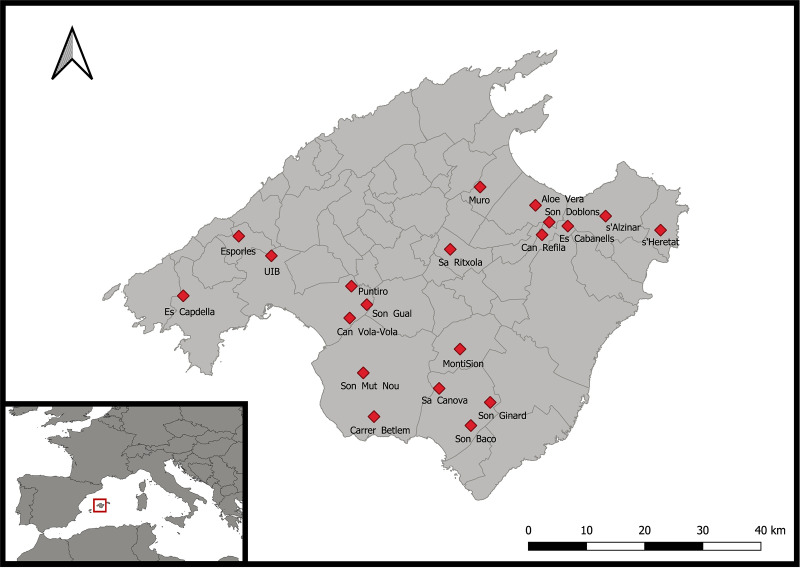
Location of the 20 study sites across Mallorca Island (Western Mediterranean Basin, Spain). The inset represents the western part of the European continent.

We selected 20 wild *Olea europea* communities (“9320 *Olea* and *Ceratonia* forests” habitat type from the EU Habitats Directive 92/43/EEC) within Mallorca Island (study sites, hereafter; Fig 1), by means of aerial photography and using the SIOSE database (Spanish acronym for *Soil Occupation Information System of Spain*, [38]). These communities were chosen to be within agricultural landscapes (not very close to urban areas or to the sea) and to differ in the composition and heterogeneity of their surrounding landscape. Study sites were separated between ca. 2 and 83 km, with an average distance between closest pairs of 6.5 ± 3 km. All communities were located in the lowland, with verage elevation of 98.4 ± 12.5 m asl (range: 29–250) and annual precipitation of 575.5 ± 37.9 mm (range: 311.6–931.4; Balears Meteo climatic network [39]), acumulated from the autumn previous to our sampling, which is the crucial period for seedling recruitment in Mediterranean therophytic grasslands [40]. We selected *Olea europea* communities because they are species-rich communities that are widespread across the island and appear in landscapes differing in heterogeneity and composition. By always selecting the same type of communities in areas with homogeneous topography and precipitation regimes, our variation among sites might represent well the variation along the landscape gradient. In each study *Olea europaea* community, we intensively sampled a large area of 1 hectare, to avoid potential sampling biases related to within-site variability in abiotic conditions (such as microtopography, soil type and humidity) that could cause microsite variation in species composition. The study was conducted on private lands, and the owners allowed our work on their properties. No specific permissions were required as the study was not performed in protected areas and did not involve endangered species.

### Landscape characteristics

We used ArcMap version 10.5 [41] and land-use information from the last update of SIOSE database (year 2014) [38] to describe the landscape characteristics of the 20 study sites. We stablished a 1-km buffer around the centre of each study site, and estimated the area covered by different land-cover classes in this buffer zone. In total, we had 19 land-cover classes in the buffer zones, including different natural and semi-natural habitats (mainly conifer, mixed and hardwood forests, transition woodlands, sclerophyllous shrubs, rocky zones, pastureland; 7 classes), crops (mainly herbs and tree cultivars; 5 classes) and artificial areas (7 classes). Although there were some small artificial areas in the landscape (mainly small roads or trails, buildings and artificial green areas or water sheets), we sampled on agricultural landscapes, avioiding important urban areas in the surrounding of our study sites to minimize the extent of other landscape perturbations different to agricultural uses; thus, artificial areas only corresponded to 6.01% ± 1.4 of the buffer zones.

With these data we calculated the percentage of natural area and crops, as descriptors of landscape composition. We measured the percentage of area within the buffer occupied by natural and semi-natural areas, and by crops. The percentage of landscape occupied by natural or crop areas was calculated as: (total area of natural or crop habitats / total area in the buffer zone) × 100. In addition, we calculated landscape heterogeneity, as the diversity of all the land-cover classes in the buffer zones, calculated as Shannon’s [42] diversity index. Same as for the plants, the diversity of landscape cover-layers was calculated using the R-package *vegan* [43].

In previous analyses, we also estimated the variables using 2-km buffer zones around the centre of the study sites (not shown), but the models performed overall better (lower values of corrected Akaike Information Criteria (AICc)) with the data at 1 km, and therefore, we report these results here. However, the landscape was roughly the same at 1 and 2 km, as shown by the highly significant correlations of the variables at the two scales (all P-values < 0.0005; r = 0.85, 0.88 and 0.71, for percentage of natural habitats, crops and landscape heterogeneity respectively).

### Field surveys

#### Plant richness

At the beginning of 2018, in each of the 20 *Olea europaea* communities, we established a 100 × 100 m permanent plot where we performed a complete inventory of vascular plant species. In each study site, we performed a survey three times throughout the year (in early-spring, late-spring and mid-autumn) to ensure that we found all the plant species present, and that we did not understimate the floristic composition due to the high intra-annual variability typical of these Mediterranean communities [44]. We spent between two and three hours on each floristic survey at each study site. Most plant species were identified in the field, while samples of species with complex taxonomical features were taken to the laboratory for accurate determination. All samples were identified at species or subspecies level, except for a few cases (1.3%) in which there was just taxonomical information at the genus level (specifically, *Allium*, *Bromus*, *Carex*, *Ophrys* and *Orobanche*). To simplify, we use the term species instead of taxa throughout the manuscript; thus, species richness includes taxa both at the subspecies and genus level. Nomenclature followed the Plant List [45], with the exceptions of *Arum pictum* subsp. *sagittifolium* Rosselló & L. Sáez, *Hedypnois cretica* subsp. *monspeliensis* Murb., *Hyparrhenia sinaica* (Delile) Llauradó ex G. López, *Ophrys bertolonii* subsp. *balearica* (P. Delforge) L. Sáez & Rosselló, *Oprhys fusca* subsp. *bilunulata* (Risso) Aldasoro & L. Sáez, *Scorpiurus sulcatus* L., and *Selaginella denticulata* (L.) Spring, for which we followed Gil and Llorens [46] in order to maintain those taxonomical identities.

#### Plant density

We estimated plant species density at each study site in spring 2018, coinciding with the flowering peak of the study community. Plant density was estimated for each species as the total number of records in 100 m linear transects located in the middle of the sampling 1-ha plots. For that, we recorded the presence of each species every 25 cm along the transect (in total 400 sampling points at each site). Thus, the frequency of each plant species was calculated as the number of records for this species along the transect. Those plant species that appeared in the vegetation survey but that did not appear in the 100 m transect were assigned a frequency of 0.5. In this way, these species were taken into account for diversity analyses, but were given a lower abundance than the minimum abundance recorded (1 record/transect) in the density surveys.

### Plant taxonomical diversity

To describe the plant taxonomical diversity at each of the 20 study sites, we calculated plant richness, as the total number of plant species recorded in the richness surveys. We first calculated plant richness for the complete set of species, and second by categorizing the species into those typical of *Olea europaea* communities versus ruderal/segetal species. Ruderal species are those associated with disturbed habitats and segetal species are those occurring in crop fields, and they were combined for statistical analyses (ruderal species, hereafter; habitat preference defined following de Bolòs et al. [47] and our own experience). The categorization into ruderal and non-ruderal species was conducted to assess whether any potential peak in plant richness along the gradients of heterogeneity and crops in the landscape was due to the colonization by ruderal species.

Using the data recorded in the density surveys at each site, we also calculated plant diversity, as the Shannon’s [42] diversity index, and plant evenness, as Pielou’s [48] evenness index, calculated as J = H'/ln(S), where H' is Shannon diversity and S is species richness. J varies from 0 to 1, and it is lower when there is dominance of one species.

### Local contribution to beta diversity

To understand how the landscape influenced composition uniqueness and the extent to which each site contributed to regional beta diversity, we used the index of local contribution to beta diversity (LCBD; [49]). LCBD was estimated for each site based on quantified diversity data with the function *beta*.*div* from the R-package *adespatial* [50] with 999 permutations and a *hellinger* transformation, which is the most appropriate for the study of beta diversity [49].

### Species functional traits

For all the plant species found in the communities at the 20 study sites, we compiled data on 11 functional traits related to their life form, physiology and reproduction (Table 1). Trait data were compiled from the following plant databases: BROT [51], LEDA [52] and TRY [53], as well as from published floras [47, 54], and our own data. We describe below the traits selected for the analyses.

**Table 1:** Functional traits compiled for the plant species in the study sites.

Group	Trait	% data^a^	Functional attributes	Reference source
Life traits	Life form	100.0	Chamaephyte, geophyte, hemicryptophyte, liana, macrophanerophyte, nanophanerophyte, therophyte	[47]
	Life span	73.6	Very short (<2 yr), short (2–5 yr), medium (5–25 yr), long (25–150 yr), very long (>150)	[51–53]
Physiological traits	Plant height	98.7	Numerical value (m)	[47, 51–53]
	Leaf area	99.0	Very small (<25 mm^2^), small (25–225 mm^2^), medium (225–2025 mm^2^), large (2025–4550 mm^2^), very large (>4550 mm^2^)	[47, 51–53]
	Specific leaf area (SLA)	58.9	Numerical value (mm^2^/mg)	[51–53]
Reproductive traits	Clonality	100.0	No (without clonal ability), yes (clonal plant)	[47, 51–53]
	Type of floral unit	100.0	Apetalous, flowers, pseudanthium, fern.	[47, 54]
	Pollination syndrome	100.0	Entomogamous (1), non-entomogamous (0)	[53], self knowledge
	Annual seed production	99.5	Rare (rarely, if ever, produces seeds in the study area), few (<50), medium (50–500), many (>500)	[51–53]
	Seed mass	64.7	Very light (<0.5 mg), light (0.5–1.5 mg), medium (1.5–10 mg), heavy (>10 mg)	[51–53]
	Dispersal mode	100.0	Zoochory (1), without zoochory (0)	[51–53]

^a^ Percentage of species for which information was available.

#### Life traits

We categorized the species according to their life form, i.e., the morpho-biological structure used for environmental adaptation, and to their life span, that indicates species longevity. (i) For life form, species were categorized as chamaephyte, geophyte, hemicryptophyte, liana, macrophanerophyte, nanophanerophyte, and therophyte following the classification of Raunkiær [55]. (ii) For life span, species were categorized as very short (<2 yr), short (2–5 yr), medium (5–25 yr), long (25–150 yr), and very long (>150) lived species.

#### Physiological traits

As relevant physiological traits of plant species, we selected plant height because it determines a species' ability to compete for light [56, 57], leaf area, that reflects the photosynthetic rate, and specific leaf area (SLA), that indicates the trade-off between leaf longevity and the maximum photosynthetic rate, and thus controls both growth rate and the capacity to respond to disturbance [58, 59]. (i) Species’ average plant height (m), defined as the length between the highest photosynthetic tissue and the base of the plant, was included in our analyses as a continuous trait. (ii) For leaf area, species were categorized as very small (<25 mm^2^), small (25–225 mm^2^), medium (225–2025 mm^2^), large (2025–4550 mm^2^), and very large (>4550 mm^2^). Leaf area was given as the one-sided projected area (mm^2^) of an individual leaf excluding the petiole and leaflets area × leaflets number for compound leaves. (iii) Specific leaf area (SLA), defined as leaf area to dry weight ratio (mm^2^/mg); average SLA for each plant species was included in our analyses as a continuous trait.

#### Reproductive traits

Regarding reproductive traits, we categorized the species based on their capability of clonal reproduction, type of floral unit, pollination syndrome, annual seed production and seed mass. (i) For clonality, species were categorized as clonal or without clonal ability. (ii) For floral unit, species were categorized as apetalous, flowers, pseudanthium (inflorescences that correspond to a single flower-like structure, such as capitulum, cyathium, umbel or glomerulus), and fern. (iii) For pollination syndrome, species were categorized as entomogamous and non-entomogamous species. (iv) For annual seed production per plant, species were categorized as rare (rarely, if ever, produces seeds in the study area), few (<50), medium (50–500), many (>500). (v) Species’ average seed mass (mg) was included in our analyses as a continuous trait. (vi) For seed-dispersal mode, species were categorized as animal-dispersed species (including endozoochory, epizoochory, and mirmecochory), and abiotically-dispersed species (including gravity, anemochory, ballochory, hydrochory, and hemerochory).

For clonality and pollination syndrome, we dealt with missing values by using data of other similar species in the genus, as previously done in other studies of plant traits [60], whereas for the other traits we left the missing values as empty data (see Table 1 for information completeness).

### Functional diversity

Using the functional traits described in the previous section and the data on plant species abundance at each study site, we calculated the following indices describing plant functional diversity: (i) Functional richness, which represents the amount of functional trait range (i.e., functional space) occupied by all trait combinations represented in the community [32]; (ii) Functional evenness, which represents the homogeneity of a sample's trait distribution in the functional space [32]; (iii) Functional divergence, which is a measure of how spread-out or clustered the species are in the functional space [32]; and (iv) Functional dispersion (FDis), which is the average distance of individual species to the centroid of all species in the multidimensional functional space, and quantifies community functional specialisation [33]. FDis is little influenced by species number [33], contrary to other indices such as functional richness [32] or functional divergence [61]. Following Laliberté et al. [62], FDis was not weighted by species relative abundances because rare species may contribute substantially to resilience [61]. As the species-by-species distance matrix could not be represented in a Euclidean space, we used the “cailliez” correction method, and a minimum number of axes (m) to reduce dimensionality. All these indices were calculated using the *dbFD* function from the FD package [33, 63] in R version 3.5.0 [64].

### Statistical analyses

All the statistical analyses reported here were conducted in R version 3.5.0 [64]. We performed separate generalized linear models (GLM, library *nlme*, [65]) to study the effects of landscape composition and heterogeneity on plant richness, diversity and evenness, as well as on LCBD and the different indices of functional diversity (functional richness, evenness, divergence and dispersion). In all these models the study sites were the sampling units. Landscape heterogeneity (i.e. the Shannon diversity of landscape cover-classes), and the percentage of natural and crop habitat were included as predictor variables in the full models. As we hypothesized that the highest diversity might occur at intermediate disturbance levels [12, 13], we included the variable percentage of crops in the landscape both as a linear (% Crops, hereafter) and quadratic (% Crops^2^, hereafter) terms in the analyses. To avoid collinearity between these two terms, we first standardized the variable to μ = 0 and σ = 1 and then calculated its quadratic term [66]. We then ran variance inflation factor (VIF) analyses to identify collinear predictor variables that should be removed from further analyses (VIF value ≥ 3; [67]). The percentage of crops and natural habitats in the buffer zone were collinear, so we excluded the percentage of natural habitats from the analyses (as we were specifically interested in testing the effects of extent of agriculture in the landscape). All the other variables did not show collinearity problems. To assess whether any potential peak in plant richness along landscape gradients was due to the colonization of ruderal species, we also ran a separate model in which the interactions between habitat preference (ruderal vs the other species) and landscape characteristics (composition and heterogeneity) were tested. We used Poisson distribution with log-link function for the models of plant richness, gamma distribution with log-link for the models of functional evenness and divergence, and Gaussian distribution with identity link function for the other variables, as previous Lilliefors tests [68] indicated that these variables fulfilled the assumptions of normality. We used dredge function in R (library MuMIn; [69]) to generate the best models with combinations (subsets) of all the terms from the global model, through automated model selection. One model was considered better than other when ΔAICc > 2. We show the results of the best models in the main text and any alternative model in Supporting Information. Significance of predictor variables was based on likelihood ratio rests (LRT). To ensure that the use of GLM was adequate, we run Moran’s I tests to assess whether there was spatial autocorrelation in the data and/or the residuals of the models by using the functions *moran*.*mc* and *lm*.*morantest* (respectively) in R-package *spdep* [70, 71]. These tests indicated that there was no spatial autocorrelation either in the data or in the residuals (S1 Table) and, therefore, the use of GLMs was appropriate.

## Results

In total, we recorded 397 plant species and 63 families in the 20 study sites, with an average of 120.85 ± 4.37 species (range: 75–156) and 36.50 ± 0.92 families (range: 30–45) per study site. Species were distributed according to their life form as follow: 216 (54.41%) therophytes, 55 (13.85%) hemicryptophytes, 47 (11.84%) geophytes, 36 (9.07%) chamaephytes, 18 (4.53%) nanophanerophytes, 17 (4.28%) macrophanerophytes, and 8 (2.02%) lianas. Taxonomical identities of all the plant species included in the study, as well as their compiled traits, are available at S2 Table. Data on taxonomical and functional diversity, and landscape composition and heterogeneity for each study site are available at S3 Table.

### Taxonomical diversity along landscape gradients

**Fig 2:**
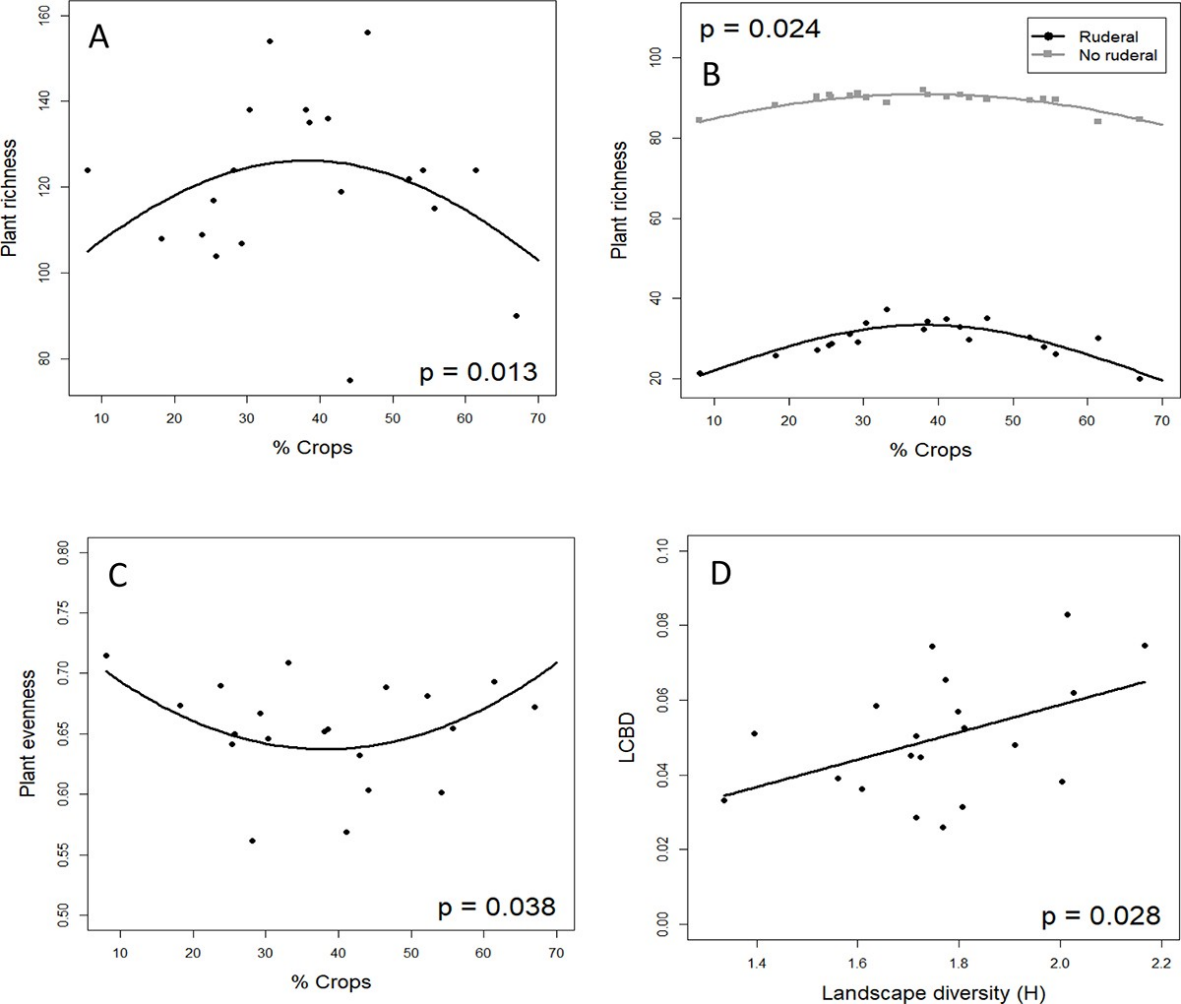
Relationship between plant taxonomical diversity and landscape characteristics in agricultural landscapes. (A) Plant richness and the percentage of crops in the surrounding landscape; (B) plant richness separately for ruderal and non-ruderal species and the percentage of crops in the surrounding landscape; (C) plant evenness and the percentage of crops in the surrounding landscape; and (D) local contribution to beta diversity (LCBD) and landscape heterogeneity. Lines represent the estimates for the best models and the dots the values for each study site.

Plant richness peaked at intermediate percentage of crops in the surrounding landscape (Table 2A; Fig 2A). When we tested the interaction between the percentage of crops and the ruderal character of species, we found this interaction to be significant (Habitat preference -ruderal vs. non-ruderal-: χ^2^ = 299.62; df = 1; P-value < 0.0001; Habitat preference × % Crops^2^: χ^2^ = 5.099; df = 1; P-value = 0.024), which indicates that the increase in richness at intermediate percentage of crops was mostly due to the increase of ruderal species, although the non-ruderal plants also peaked at intermediate crop levels (Fig 2B). Contrary to plant richness, plant evenness was minimum at intermediate percentage of crops in the landscape (Table 2B; Fig 2C), and as a consequence, Shannon’s plant diversity was not significantly influenced by any of the variables tested (Table 2C). For plant evenness, we found two alternative models (ΔAICc > 2) that included landscape heterogeneity, either alone or together with the quadratic estimate of percentage of crops in the surrounding landscape (S4A Table), but this new variable was non-significant.

**Table 2:** Best models showing the relationships between different indices describing plant diversity and landscape characteristics.

Model	Variable	χ^2^	df	P-value ^a^
A) Plant richness	% Crops^2^	6.16	1	0.013
B) Plant evenness	% Crops^2^	4.32	1	0.038
C) Plant diversity (H’)	% Crops^2^	1.05	1	0.305
D) LCBD	Landscape heterogeneity	4.8182	1	0.028

LCBD, Local Contribution to Beta Diversity; % Crops^2^, squared percentage of crops.

^a^ P-values are based on likelihood ratio tests (LRT).

### Local contribution to beta diversity along landscape gradients

LCBD indices for the different study sites varied from 0.029 to 0.083, and were significant for four localities (LCBD > 0.065; Es Cabanells, Muro, s’Heretat and UIB). LCBD increased significantly with Landscape heterogeneity (Table 2D; Fig 2D). An alternative model (ΔAICc > 2) to the best one also included the quadratic estimate of percentage of crops in the surrounding landscape (S4B Table), but its effect was non-significant.

### Functional diversity along landscape gradients

**Fig 3:**
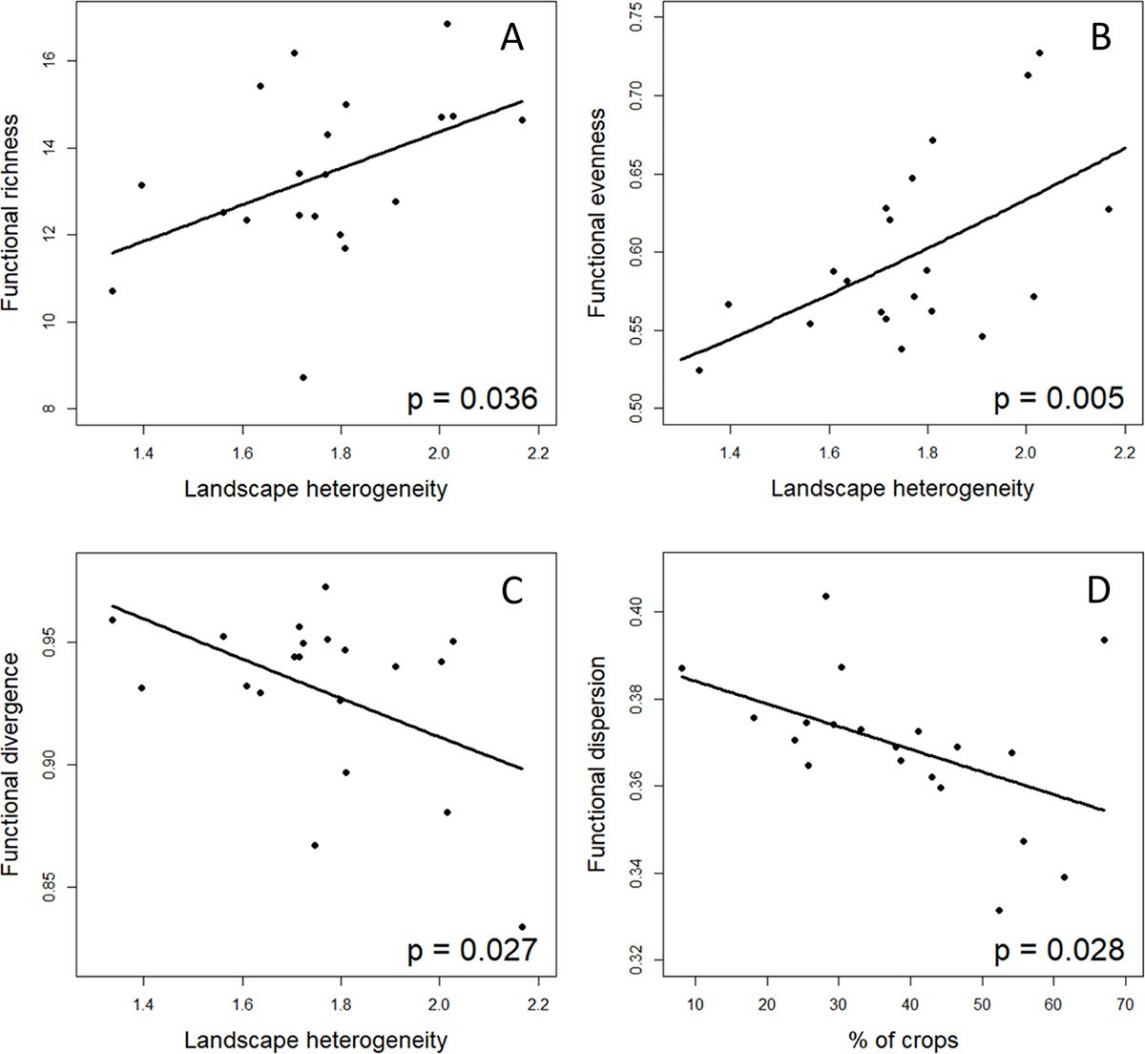
Relationships between functional diversity and landscape characteristics in agricultural landscapes. (A) Functional richness and landscape heterogeneity; (B) functional evenness and landscape heterogeneity; (C) functional divergence and landscape heterogeneity; and (D) functional dispersion and the percentage of crops in the surrounding landscape. Lines represent the estimates for the best models and the dots the values for each study site.

Best models for functional diversity indices indicated that functional richness and evenness increased with landscape heterogeneity (Table 3A and 3B; Fig 3A and 3B), whereas functional divergence decreased with this variable (Table 3C; Fig 3C). Functional dispersion, however, was negatively related to the percentage of crops in the landscape (Table 3D; Fig 3D). For functional divergence, we found an alternative model (ΔAICc > 2) that also included the quadratic estimate of percentage of crops in the surrounding landscape (S4C Table), but with a non-significant effect.

**Table 3:** Best models showing the relationships between different indices describing functional diversity and landscape characteristics.

Model	Variable	χ^2^	df	P-value ^a^
A) Functional richness	Landscape heterogeneity	4.407	1	**0.036**
B) Functional evenness	Landscape heterogeneity	8.005	1	**0.005**
C) Functional divergence	Landscape heterogeneity	4.857	1	**0.027**
D) Functional dispersion	% Crops	4.811	1	**0.028**

^a^ P-**values** are based on likelihood ratio tests (LRT).

## Discussion

In this study, we show that an intermediate percentage of crops in the landscape increases the taxonomical richness of plant communities, supporting the Intermediate Distrubance Hypothesis (IDH). However, this richness peak at intermediate levels of agriculture is mostly due to the appearance of ruderal species in the communities, which may explain why plant evenness decreases at intermediate crop levels. In addition, we show that local contribution to beta diversity as well as functional richness and evenness increased with landscape heterogeneity, indicating that the heterogeneity of Mediterranean landscapes contributes to maintaining these rich ecosystems through both taxonomial and functional diversity. Interestingly, communities within highly heterogeneous landscapes also showed low functional divergence (i.e., high functional redundancy), suggesting a potential further positive effect of landscape heterogeneity on community resilience against disturbances. However, a high percentage of agriculture in the landscape seems to act as an environmental filter that reduce functional dispersion, which could have overall negative effects on ecosystem functions.

### Plant taxonomical diversity

We have identified a total of 397 plant species from 63 families in the 20 study sites, which represents high diversity values in the context of the general flora of the Balearic Islands (in total 1729 plant species and 122 families, [35]). The *Olea europaea* plant community consists of woodlands and sclerophyllous shrublands characteristics of the Majorcan thermomediterranean dry-subhumid and dry-semiarid series of vegetation [37], interspersed with the therophytic grasslands which are known to hold a high biodiversity [72]. Indeed, communities in semi-open disturbed shrublands have some of the highest plant alpha diversities in the world (e.g., 138.7 species / 0.1 ha, in Northern Israel), especially in terms of annual plants [73]. This is also the case in our study system, with a high proportion (over 50%) of plant species being therophytes. In this study, we focused on the effects of landscape on plant diversity across comparable communities in a single year, Mediterranean therophytic grasslands may also experience strong inter-annual fluctuations related to meteorological conditions [40, 44, 74]. Future studies may evaluate whether the strength of the relationships between landscape characteristics and plant diversity could be affected under the climate change scenario.

Plant taxonomical richness peaked at intermediate levels of crops in the landscape. This result supports the IDH, predicting that species diversity is highest at intermediate levels of disturbance because of a balance between competitive exclusion and the establishment of dominant species [12, 13]. IDH has been empirically demonstrated for other plant communities, such as dry tropical forests [75] and riparian and upland plant communities [76], but see [20]. Our results are also in line with a study that showed higher overall beta diversity in agricultural landscapes of Central Europe compared to non-agricultural ones [77]. However, our analyses revealed that although overall species richness peaked at intermediate levels of percentage of crops in the landscape, the highest increase was related to the ruderal (both ruderal and segetal) species. Therefore, the gradient of area dedicated to agricultural activies not only results in changes in plant richness but also in changes in species composition by affecting the proportion of ruderal species. These results agree with those of Leßmeister et al. [78], who reported a change in species composition and an overall increase in species richness derived from an increase in the proportion of ruderal and segetal species in modified landscapes of West African savanna. Despite the effect of percentage of crops in the landscape on plant richness, we did not find any effect of this variable on the Shannon’s diversity of plant species. This is likely due to the fact that the peak of ruderal species at intermediate crop levels in the landscape was also related to a decrease in species evenness, explained by the low abundance of ruderal species relative to other species in the communities. Contrary to our results, higher species evenness with increasing disturbance intensity has been reported in riparian habitats, but species richness and diversity patterns are known to be context and system dependent [76].

Lastly, our results indicated that heterogeneous landscapes increase the local contribution to beta diversity of communities. Heterogeneous landscapes harbour more habitats and niches, which in turn might positively influence species richness in the study communities [23, 24]. In this work we have shown that although overall plant richness peaks at intermediate levels of crops in the landscape, it is the heterogeneity what determines how unique the communities are in terms of species. This may be explained by a higher species replacement (simultaneous increases in gains and losses of species) in heterogeneous landscapes holding different habitats due to environmental filtering and/or competition processes [79]. According to our results, Santana et al. [28] reported an increase in the spatial variation of species compositions of bird communities with the landscape heterogeneity in Mediterranean farmlands.

### Functional diversity

Functional diversity is expected to predict community response to environmental changes better than species richness [80, 81]. In our study, we found an overall positive effect of landscape heterogeneity on plant functional diversity, agreeing with earlier findings that have shown the role of landscape heterogeneity in maintaining functional diversity in hedgerow networks of agricultural landscapes in Western France [82]. Here, both functional richness and evenness were highest when the communities were surrounded by more heterogeneous landscapes. This positive relationship between functional richness and heterogeneity was expected, because diverse landscapes containing more habitats may increase the probability of different species with varying functional traits to colonize their communities [83, 84]. Indeed, this may be the case in our study sites, as we have shown a positive relationship between landscape heterogeneity and local contribution to beta diversity. Interestingly, in our communities, variations in functional diversity seem to be more related to the inclusion of new unique species in communities (LCBD) along the heterogeneity gradient than with overall total richness, which peaked at the intermediate crop level. The reason might be that increases in the number of ruderal species at intermediate disturbance gradients did not involve increasing effective functional diversity. Consequently, it seems to be landscape heterogeneity what defines effective taxonomical and functional richness in this Mediterranean ecosystem. It has been argued that as functional richness depends on taxonomical diversity and does not account for species abundance, its values are highly susceptible to rare and extreme traits in the community, which might lead to misinterpretations of the functional capacity of communities [32, 33, 80]. However, we found a similar positive relationship between landscape heterogeneity surrounding *Olea europaea* communities and functional evenness, i.e., effective use of functional space. This indicates that a high diversity of habitats in the landscape enhances the addition of traits to communities in equal abundances, so heterogeneous landscapes are not only functionally richer but also their traits are more homogenously represented. Opposite to these positive relationships, we found that functional divergence, which describes the abundance distribution of traits in the comunity, decreased with landscape heterogeneity. A low functional divergence indicates that the most abundant species have traits more similar to the centre of the functional trait range [32], and therefore, dominant species might have a higher trait overlap [80]. Some studies have interpreted a low functional divergence in terms of decreased ecosystem function due to less efficient resource use [85]. However, higher trait overlap implies an increase in functional redundancy (i.e., higher number of species contributing to an ecosystem function), which overall may have positive effects on community resilience against disturbances [86, 87].

Finally, functional dispersion, an index that indicates clustering in the multidimensional trait space and that is independent of both plant richness and abundance [33], decreased with the extent of agriculture in the landscape. A reduction in functional dispersion translates to a higher functional specialisation, i.e. the communities contain species having more similar functional traits [33]. Thus, our results show that large areas of landscape dedicated to agricultural activities act as a strong environmental filter [82, 88] that only allow the presence of functionally similar species in the communities. Such a reduction in functional dispersion with the extension of crops in the landscape may lead to a decrease in ecosystem functions, because communities might lose the capability to respond to environmental changes or disturbance [86, 87]. Thus, this study emphasises the importance of taking into consideration several indices of both taxonomical and functional diversity to deeply understand the complex relationships between changes in landscape composition and heterogeneity and ecosystem functions.

### Conclusions

Our study indicates that the heterogeneity of extensively managed Mediterranean landscapes contributes to maintaining rich communities in terms of species and traits, which may be highly resilient against disturbances due to their high functional redundancy. Regarding the extent of agriculture in the landscape, we show that an intermediate level of crops increases the taxonomical richness of plant communities, supporting the IDH. However, this richness peak is mostly due to the appearance of scarce ruderal species in the communities, without further effects on functional diversity. Indeed, a large extent of agriculture in the landscape seems to act as a strong environmental filter that reduces functional dispersion, which may have overall negative effects on ecosystem functions. Our study highlights the benefit of maintaining low to moderate levels of extensive agriculture and favouring landscape heterogeneity to preserve the complexity, biodiversity and functionality of the species-rich *Olea europaea* communities.

## Supporting information

S1 Table Results of Moran’s I tests to evaluate the existence of spatial autocorrelation in the data and the residuals of the models. (DOCX)

S2 Table Floristic inventory for each study site and the compiled traits associated to them. (XLSX)

S3 Table Coordinates, taxonomical and functional diversity, and landscape composition and heterogeneity for each study site. (XLSX)

S4 Table Best and alternative models (ΔAICc > 2) for plant evenness, local contribution to beta diversity and functional divergence. (DOCX)
