# Supplementary material for: The role of landscape composition and heterogeneity on the taxonomical and functional diversity of Mediterranean plant communities in agricultural landscapes
Source: PLoS One. 2020 Sep 16;15(9):e0238222. doi: 10.1371/journal.pone.0238222 (PMC7494112; doi:10.1371/journal.pone.0238222)
Supplement: S1 Table — (DOCX) [file pone.0238222.s001.docx]

**S1 Table. Results of Moran’s I Tests to Evaluate the Existence of Spatial Autocorrelation in the Data and the Residuals of the Models.**

| **Model** | **Variable** | **Moran I** | **P-value** |
| --- | --- | --- | --- |
| A) Plant richness | Data | 0.088 | 0.307 |
|  | Model residuals | 0.100 | 0.460 |
|  | Data ruderals/non-ruderals | -0.736/0.157 | 0.999/0.199 |
| B) Plant evenness | Data | 0.040 | 0.356 |
|  | Model residuals | 1.246 | 0.106 |
| C) Plant diversity (H’) | Data | 0.148 | 0.238 |
|  | Model residuals | 1.124 | 0.131 |
| D) Local Contribution to Beta Diversity (LCBD) | Data | 0.124 | 0.263 |
|  | Model residuals | -0.531 | 0.702 |
| E) Functional richness | Data | 0.319 | 0.080 |
|  | Model residuals | 1.118 | 0.132 |
| F) Functional evenness | Data | 0.459 | 0.078 |
|  | Model residuals | -0.022 | 0.509 |
| G) Functional divergence | Data | -0.007 | 0.366 |
|  | Model residuals | -0.741 | 0.771 |
| H) Functional dispersion | Data | -0.380 | 0.886 |
|  | Model residuals | -0.516 | 0.697 |
