# Supplementary material for: The role of landscape composition and heterogeneity on the taxonomical and functional diversity of Mediterranean plant communities in agricultural landscapes
Source: PLoS One. 2020 Sep 16;15(9):e0238222. doi: 10.1371/journal.pone.0238222 (PMC7494112; doi:10.1371/journal.pone.0238222)
Supplement: S4 Table — (DOCX) [file pone.0238222.s004.docx]

**S4 Table. Best and Alternative Models (ΔAICc > 2) for Plant Evenness, Local Contribution to Beta Diversity and Functional Divergence.**

| **Response variable** | **Best and Alternative models** | **ΔAICc** |
| --- | --- | --- |
| A) Plant evenness (J) | % Crops^2^ | -67.5 |
|  | Landscape heterogeneity | -65.9 |
|  | Landscape heterogeneity + % Crops^2^ | -65.7 |
| B) Local Contribution to Beta Diversity (LCBD) | Landscape heterogeneity | -106.6 |
|  | Landscape heterogeneity + % Crops^2^ | -105.5 |
| C) Functional divergence | Landscape heterogeneity | -75.2 |
|  | Landscape heterogeneity + % Crops^2^ | -74.4 |

% Crops^2^, squared percentage of crops.
